# 1221. Genomic Factors Affecting the Efficacy of Antimicrobial Therapy in Daptomycin-, Linezolid-, Vancomycin-Resistant *Enterococcus faecium* (DLVRE)

**DOI:** 10.1093/ofid/ofab466.1413

**Published:** 2021-12-04

**Authors:** Samuel W Gatesy, Nathan B Pincus, William Justin Moore, Omar Al-Heeti, Tejas Joshi, Kelly E R Bachta

**Affiliations:** 1 Northwestern University, Libertyville, Illinois; 2 Northwestern Medicine, Chicago, IL; 3 Northwestern Feinberg School of Medicine, Chicago, Illinois

## Abstract

**Background:**

Nosocomial acquisition of vancomycin-resistant *Enterococcus* (VRE) is one of the most challenging problems in healthcare. As *Enterococcus* isolates are increasingly resistant to vancomycin, clinicians now rely on alternative antimicrobial therapies including linezolid and daptomycin (DAP) to treat infections. For multidrug-resistant (MDR) VRE, combination therapy with beta-lactams and daptomycin has been shown to be effective.

**Methods:**

Following initiation of empiric DAP and ceftaroline (CPT) for an MDR *E. faecium* bloodstream infection (VRE_001), we aimed to determine if there existed *in vitro* synergy between both agents that supported their clinical use. Combination synergy testing was performed using E-test strips and minimal inhibitory concentrations (MICs) were read at 24 hours. For whole genome sequence-based analysis (WGS), genomic DNA from VRE_001 was used for both short read (Illumina MiSeq) and long-read sequencing (MinION, Nanopore). The complete genome was assembled and the NCBI AMRFinderPlus program used to identify known resistance mechanisms.

**Results:**

Original MICs of VRE_001 from the clinical microbiology laboratory at Northwestern Memorial revealed an MDR *E. faecium* (Table 1). Combination synergy testing in the experimental laboratory revealed only modest amounts of synergy between CPT and DAP (Table 2). Following WGS, VRE_001 was identified as an ST-584 *E. faecium* with a 3.2 Mbp genome, including a single chromosome and five plasmids. WGS analysis revealed several mechanisms of antimicrobial resistance (Table 3) genetically supporting the observed MDR-DLVRE phenotype.

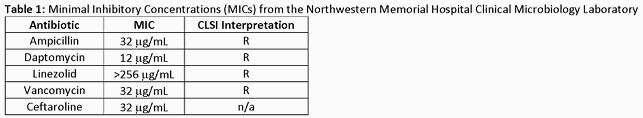

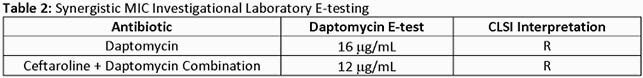

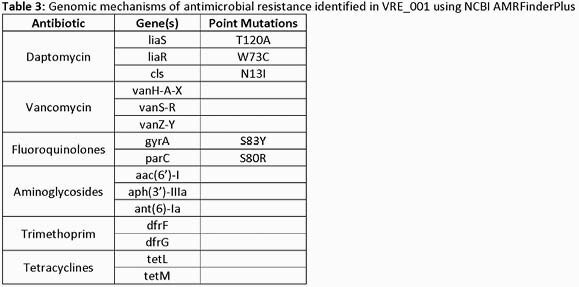

**Conclusion:**

Our investigational antimicrobial testing allowed for real-time *in vitro* analysis of synergistic MICs in a case of DLVRE bacteremia. Despite the fact that *in vitro* testing of CPT and DAP did not support the clinical usage of combination antimicrobial therapy, the patient cleared their blood cultures. WGS of VRE_001 revealed a plethora of antimicrobial resistance mechanisms including three mutations that explain high levels of DAP resistance. Synergy testing is not routinely available in most clinical laboratories, but rapid implementation of investigational MIC testing paired with genomic analysis may one day successfully support real-time clinical decision making.

**Disclosures:**

**All Authors**: No reported disclosures

